# Parathyroid score can predict the duration of required calcium supplementation after total thyroidectomy

**DOI:** 10.1371/journal.pone.0174088

**Published:** 2017-03-28

**Authors:** Bup-Woo Kim, Soo Young Kim, Yong Sang Lee, Seok-Mo Kim, Hang-Seok Chang, Cheong Soo Park

**Affiliations:** Department of Surgery, Gangnam Severance Hospital, Yonsei University College of Medicine, Seoul, Republic of Korea; CHA University, REPUBLIC OF KOREA

## Abstract

**Background:**

Postoperative hypoparathyroidism is the most common complication after total thyroidectomy, owing to unintentional injury or decreased blood flow to the parathyroid glands. Prediction of postoperative hypoparathyroidism would be helpful for surgeons to manage postoperative hypocalcemia. In this study, we scored the discoloration of the parathyroid glands using a new parathyroid scoring system and evaluated the correlation between the parathyroid score and duration of required calcium supplementation after total thyroidectomy.

**Methods:**

A total of 316 patients undergoing total thyroidectomy between November 2009 and April 2010 were enrolled in this retrospective study. Parathyroid scoring was performed by one experienced surgeon. The status of each of the 4 parathyroid glands was classified as normal color (3 points), slightly discolored (2 points), dark discoloration (1 point), or loss of the gland (0 points), resulting in possible total scores of 0–12. Serum parathyroid hormone (PTH), serum calcium, and ionized calcium concentrations were measured at 2 hours, 2 weeks, 3 months, 6 months, and 1 year after surgery. Patients were also divided into three groups based on the duration of required calcium supplementation: no required supplementation (n = 260, 82.3%), required supplementation for <6 months (n = 38, 12%), and required supplementation for ≥6 months (n = 18, 5.75%).

**Results:**

Parathyroid scores were positively correlated with ionized PTH concentrations at 2 hours (*r* = 0.053, p < 0.001), 2 weeks (*r* = 0.056, p < 0.001), 3 months (r = 0.032, p<0.001), 6 months (*r* = 0.072, p < 0.001), and 1 year (*r* = 0.071, p < 0.001) after thyroidectomy. Parathyroid scores were significantly and inversely associated with the duration of required calcium supplementation (p = 0.001).

**Conclusions:**

Parathyroid scores at the end of surgery might be helpful for predicting the degree of postoperative hypocalcemia after total thyroidetomy.

## Introduction

Postoperative hypoparathyroidism is the most common complication after total thyroidectomy, owing to unintentional injury or decreased blood flow to the parathyroid glands. Because the thyroid glands and parathyroid glands play very important roles in maintaining homeostasis in the body, they should remain intact. However, when total thyroidectomy is necessary because of thyroid cancer, it is important to completely remove the malignant portion while avoiding damage to the remaining portion. Hypoparathyroidism is a major cause of hypocalcemia, lengthens hospital stays, and necessitates repetitive laboratory tests and continued administration of both calcium and vitamin D [1, 2, 3].

Although the diverse causes of hypocalcemia include excessive discharge of calcium into urine due to surgical stress, hungry bone syndrome due to hyperthyroidism or hypercalcitoninemia, and vitamin D deficiency, the most important causes include physical damage to the four parathyroid glands, which are adjacent to the thyroid glands, during a total thyroidectomy; disturbance of the blood supply to the parathyroid glands resulting from damage to the blood vessels; and removal of the parathyroid glands by unintentional resection [4,5]. Therefore, an anatomical understanding of the thyroid glands and parathyroid glands in addition to meticulous surgical techniques are required to prevent these causes of hypoparathyroidism; although specialized surgeons make fewer mistakes with regard to damage to the glands, they do still occur [6,7].

After a total thyroidectomy, hypocalcemia reportedly occurs in approximately 10–50% of patients, and approximately 0.5–2% of these patients have permanent hypocalcemia that persists for at least one year [1, 8–12]. Side effects of total thyroidectomy-induced hypocalcemia include Chvostek’s sign, Trousseau’s sign, numbness, and paresthesia of the fingertips; these cause discomfort for patients and reluctance to be discharged early. Long-term progressive changes can result in abnormal skeletal microstructures [13,14].

Prediction of the degree of hypoparathyroidism, presence of hypocalcemia, and duration of required calcium supplementation through simple methods such as monitoring the color of the parathyroid glands during surgery could assist with patient management. Therefore, the purpose of the present study was to categorize the condition of the parathyroid gland using a simple scoring system immediately after a total thyroidectomy and determine the correlation between post-operative intact parathyroid hormone (iPTH) levels and duration of required calcium supplementation.

## Materials and methods

### Patients and study design

Between November 2009 and April 2010, 316 patients who underwent a total thyroidectomy with central node dissection (CND) in the Department of Surgery, Gangnam Severance Hospital, Yonsei University College of Medicine in Seoul, Korea were enrolled in this retrospective study. An ipsilateral CND (pretracheal and ipsilateral paratracheal node dissection) was performed routinely for prophylactic or therapeutic purposes, and a bilateral CND was performed for patients with bilateral thyroid cancer. To maintain the same protocol, the same experienced surgeon performed all operations and evaluations of the status of the parathyroid glands.

The subjects were divided into three groups based on the duration of calcium replacement: group I did not require calcium and vitamin D supplements or required them for ≤2 weeks; group II required supplementation for ≤6 months; and group III required supplementation for ≥6 months (Table 1). In the patients who recovered from hypoparathyroidism, calcium and vitamin D replacement was stopped.

**Table 1:** Analysis of risk factors affecting the duration of calcium requirement.

	Oral medication			*p* value
	Group I	Group II	Group III	
**Number of patients (%)**	260(82.3)	38(12.0)	18(5.75)	
**Age, mean**	19-77(45.50)	22-65(46.34)	30-65(45.89)	0.911
**Sex**				0.303
Female (%)	204(78.5)	33(86.8)	16(88.9)	
Male (%)	56(21.)	5(13.2)	2(11.1)	
**Largest primary tumor diameter in cm**	0.899(0.1–3)*	0.942(0.4–2.5)*	1.058(0.2–4.0)*	0.486
**Extrathyroidal extension (%)**	147(56.8)	19(51.4)	9(52.9)	0.799
**Multifocality (%)**	73(28.1)	15(39.5)	5(27.8)	0.516
**Central nodal status (mean)**				
dissected lymph nodes	0-18(5.62)*	1-20(6.37)*	1-15(7.00)*	0.259
metastatic lymph nodes	0-13(1.27)*	0-5(0.97)*	0-9(1.50)*	0.632
**Thyroiditis**				0.175
Yes	89(34.2)	10(27.0)	9(52.9)	
No	171(65.8)	27(73.0)	8(52.9)	
**MRND**				0.692
** Yes**	28(10.8)	5(13.2)	1(5.6)	
** No**	232(89.2)	33(86.8)	17(94.4)	
**Parathyroid score (mean)**	**7.32**	**6.45**	**4.89**	**<0.001**

Abbreviation: MRND, modified radical neck dissection.

*Data are given as mean ±SD (range)

This study was approved by the Institutional Review Board of Severance Hospital, Seoul, Republic of Korea (IRB No. 3-2016-0062). Written informed consent was waived and patient information was anonymized and deindentified prior to analysis.

### Blood tests

Serum calcium levels were measured at the same time points, and postoperative symptomatic hypocalcemia was defined as serum calcium <8.2 mg/dL or the presence of any signs or symptoms such as numbness and paresthesia of the fingertips, toes, and perioral area; Chvostek’s sign; Trousseau’s sign; or tetany. Calcium replacement was performed when serum calcium levels were <8.2 mg/dL (normal range, 8.2–10.2 mg/dL) [15].

iPTH was measured using a standard radio immunometric assay (reference range, 15–65 pg/mL; Nichols Institute, San Clemente, CA) at 2 hours, 2 weeks, 3 months, 6 months, and 1 year post-thyroidectomy.

### Assessment of parathyroid status/parathyroid scoring

**Fig 1:**
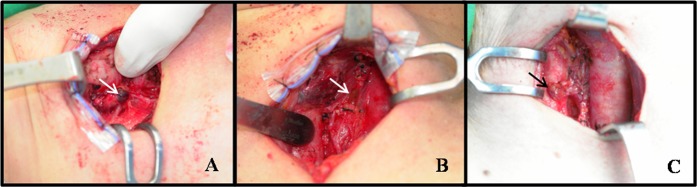
Standard examples of parathyroid scoring (PS) based on parathyroid color after total thyroidectomy. Dark discolored parathyroid (PS-1) (A); slightly discolored parathyroid (PS-2) (B); and well-preserved parathyroid color, similar to the pre-operative status (PS-3) (C).

Based on our experience with several total thyroidectomies, we developed a method of scoring the condition of the parathyroid glands after surgery; we had observed postoperative changes in the color of the glands. Therefore, it might be possible to predict damage to the parathyroid glands and postoperative need for calcium supplementation based on color. This parathyroid scoring was performed postoperatively and classified as follows (Fig 1): 3 points were assigned when the parathyroid was a normal color (yellow like a sea urchin’s egg); 2 points were assigned for a slightly discolored parathyroid (red color, without the normal yellow color, and less dark than normal thyroid gland); 1 point was assigned for a dark red (darker than the color of thyroid gland); and 0 points for a missing parathyroid after thyroidectomy. The scoring based on color was determined immediately after specimen delivery by one experienced surgeon.

### Statistical analysis

Descriptive statistics were used for the basic characteristics of the three groups. The variables included age, sex, tumor size, total number of dissected lymph nodes, total number of metastatic lymph nodes, extracapsular invasion, multifocality, and TNM stage. Comparisons between the groups were conducted using one-way ANOVA for continuous variables and Pearson’s Chi square and Fisher’s exact tests for categorical variables.

Pearson’s correlation analysis was used to assess relationships between parathyroid scores and postoperative iPTH at each of the time points post-thyroidectomy (2 hours, 2 weeks, 3 months, 6 months, and 1 year). SPSS (version 13.0; SPSS Inc, Chicago, IL) was used for all analyses.

## Results

Group I consisted of 217 patients (68%) who did not experience transient hypoparathyroidism and 43 patients (13.5%) who required calcium and vitamin D supplementation for ≤2 weeks. In group III, 18 patients (5.75%) who had permanent hypoparathyroidism for at ≥6 months, 6 patients (1.9%) recovered from hypoparathyroidism after 1 year; of the remaining 12 patients (3.8%), 2 patients (0.6%) recovered from hypoparathyroidism in the third year after surgery.

As shown in Table 1, 3 groups were compared clinicopathological information, but only postoperative parathyroid scores were significantly different (p < 0.001) between the 3 groups (Table 1).

Within each group, iPTH levels decreased with increasing duration of calcium supplementation, at 2 hours, 2 weeks, 6 months, and 1 year after surgery (Table 2).

**Table 2:** iPTH and parathyroid score according to Ca+ medication.

	Oral medication			*p* value
	Group I	Group II	Group III	
**Pre-OP iPTH (mean)**	46.35	40.96	40.27	0.075
**iPTH: 2 hours (mean)**	16.80	5.03	4.21	<0.001
**iPTH: 2 weeks (mean)**	32.47	11.02	7.89	<0.001
**iPTH: 6 months (mean)**	38.59	28.08	7.91	<0.001
**iPTH: 1 year (mean)**	32.55	24.77	12.56	<0.001

Abbreviation: Pre-OP, Pre- operation: iPTH, intact parathyroid hormone

**Fig 2:**
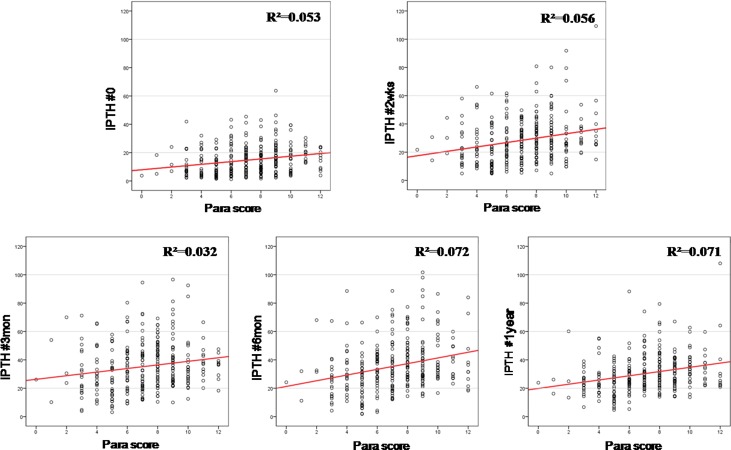
Pearson’s correlation coefficients for serum intact parathyroid hormone (iPTH) and parathyroid score (possible total scores, 0–12 points) at each post-operative measurement.

There were positive correlations between parathyroid scores and iPTH levels at all of the postoperative measurement time point, with Pearson correlation coefficient values ranging between 0.230 (at 2 hours) and 0.269 (at 6 months) (all, p = 0.001, data not shown). These correlations were clear on the scatter diagrams, despite different score ranges (Fig 2).

## Discussion

The scoring method (parathyroid score) using color that we developed to determine the status of the parathyroid glands after total thyroidectomy was able to represent the condition of the parathyroid well, as indicated by the association between the duration of calcium supplementation and iPTH values. Furthermore, the scores were correlated with iPTH levels. Therefore, the scores could be a good indicator for postoperative hypocalcemia. Hypocalcemia remains one of the most frequent complications after a total thyroidectomy, in addition to postoperative hematoma and hoarseness. Hypocalcemia occurs due to unintentional damage or decreased blood flow to the parathyroid glands [4] and can cause complaints of a tingly sensation and muscle cramping. Therefore, an important part of post-thyroidectomy management involves the continuous monitoring of calcium levels and supplementation of calcium immediately after hypoparathyroidism is identified. However, it is important to avoid conventional blood tests in patients with no possibility of hypocalcemia.

If damage to the parathyroid glands has unavoidably occurred during a total thyroidectomy, quick recognition is important. Even without the presence of a detached parathyroid gland, the ability to predict damage and the degree of damage by observing the condition of the parathyroid glands could be helpful for fast and accurate postoperative management. Therefore, we graded the discoloration of the parathyroid glands to determine if the degree of discoloration was correlated with the degree of postoperative hypocalcemia. The parathyroid scores had similar patterns with blood iPTH levels, which generally tend to increase over time after a total thyroidectomy (Fig 1). In a study conducted in 2010, a comparison of discolored parathyroid glands with normal-colored parathyroid glands and autotransplanted glands indicated that discolored parathyroid glands have a transient deterioration in function [16].

In addition, the four parathyroid glands do not function evenly. Even with similar colors, the amount of parathyroid hormones secreted by individual parathyroid glands could be considerably different, and large, dominant parathyroid glands could secrete relatively large amounts of hormones. Therefore, when a dominant parathyroid has been severely damaged or excised, the risk of postoperative transient hypoparathyroidism is high, even with high parathyroid scores for the remaining glands.

Methods to treat unavoidable damage to the parathyroid glands include auto-transplantation and calcium and vitamin D supplementation. Although auto-transplantation of the parathyroid glands is reportedly effective for long-term recovery of parathyroid gland function [17,18], the post-recovery parathyroid gland hormone levels were much lower with auto-transplantation of the parathyroid glands when there was no preserved parathyroid gland compared with auto-transplantation of the parathyroid glands when there was at least one preserved parathyroid gland [19].

In our study, there were 20 cases detected where autotransplantation was performed. Among those 20 patients, 14 patients were in group 1, 6 in group 2, and no patient was observed in group 3 (data not shown). Six of the 20 patients with autotransplantation needed more than 2 weeks of medication, but after 6 months, all patients reached normo-calcemia with normal intact parathyroid hormone level. Compared to this finding, 18 out of 50 patients without autotransplantation who needed more than 2 weeks of medication, medication for more than six month was needed (data not shown). Although it might not be statistically significant due to small sample size, autotransplantation helps in maintaining normal calcium level.

A limitation of the present study is the timing of the parathyroid gland scoring. The parathyroid glands gradually become discolored after surgery based on the degree of damage; because we scored the parathyroid glands immediately after surgery, the full extent of discoloration might not have occurred, and the function of the parathyroid glands might be scored higher than the original *in vivo* function. Gupta et al. [20] predicted postoperative hypocalcemia using iPTH levels; the combination of this approach and parathyroid scoring might enable more accurate prediction.

In conclusion, the need for and duration of calcium supplementation after total thyroidectomy were significantly related with the parathyroid score. Therefore, the parathyroid score, which is a simple method, might be effective for predicting and managing postoperative hypocalcemia, saving time and cost. These findings are important for surgeons.

## Supporting information

S1 File Parathyroid score raw data SPSS file. (SAV)
